# The Hospital. Nursing Section

**Published:** 1905-04-08

**Authors:** 


### The Hospital.

### Nursing Section.

Contributions for this Section of "The Hospital" should be addressed to the Editor, "The Hospital" Nursing Section, 28 & 29 Southampton Street, Strand, London, W.C.

No. 967.—Vol. XXXVIII. SATURDAY, APRIL 8, 1905.

### Notes on News from the Nursing World.

### NEW RIBBON FOR MILITARY NURSES.

His Majesty, it is notified in Army Orders, has approved of a new pattern ribbon to be worn with the badge of the Queen Alexandra's Imperial Military Nursing Service. The ribbon authorised by Army Order 125 of 1904 will no longer be worn. Non-commissioned officers and men of the Royal Army Medical Corps, who have been authorised, under an Army Order of 1904, to wear the badge and ribbon of the Imperial Military Nursing Service, will also wear the new ribbon in place of the old. A new paragraph will be inserted on page 2 of the regulations for admission to the Nursing Service 1904, to the following effect: "8a. A collar badge in white and gilded metal, or silver and silver-gilt, is to be worn on the points of the collar of the cloaks by members of all ranks."

### PARLIAMENT AND REGISTRATION.

The Select Committee appointed, on the motion of Sir A. Acland Hood, to consider the expediency of providing for the registration of nurses, consists this session of Dr. Robert Ambrose, Major Kenneth Balfour, Mr. Charles Douglas, Mr. Charles Hobhouse, Dr. Hutchinson, Mr. Mount, Lord Morpeth, Mr. Pierpoint, Mr. Tennant, Sir John Stirling-Maxwell, and Sir John Batty-Tuke. Of these, six were on the Select Committee last year, the new members being Major Balfour, Mr. Douglas, Mr. Charles Hobhouse, Dr. Hutchinson, and Mr. Mount. The Committee sat for the first time on Wednesday, Mr. Tennant being chosen chairman, and they will receive evidence on Wednesday next at 11.30, in Room 17 of the House of Commons. The two Registration Bills are still down for debate in the Parliamentary Order-book. The Nurses and Private Nursing Homes' Registration Bill was the 10th order on Tuesday, and, of course, was not reached; the Nurses' Registration Bill is down for second reading on Monday, April 17, and, equally of course, will not be discussed then. Mr. Harry Lawson has given notice of his intention to move the rejection of the Nurses' Registration Bill—if it ever comes on.

### A SIGNIFICANT OMISSION.

"Congratulations" have been offered to "the trained nurses of the State of Indiana" because their Bill for the Registration of Trained Nurses passed in the Senate last February. But we observe that no mention is made of the fact that the Bill in question provides for registration, without examination, of all nurses holding a certificate from a training school connected with a general hospital giving at least a two years' course. Congratulations would thus appear to be due to the nurses of Indiana because the promoters of registration have succeeded in stereotyping in that State a lower standard of training than is insisted upon, without registration, in other countries!

### THE DEFERRED NURSING ORDER.

It appears that the substitution of Mr. Gerald Balfour for Mr. Walter Long at the head of the Local Government Board does not necessarily mean that nothing more is to be heard of the recommendations in the report of the Departmental Committee. The clerk to the Ecclesall Bierlow Guardians was able to read at the last meeting a letter from the Local Government Board, stating that they "hoped to issue an Order dealing with certain of the matters which formed the subject of recommendations in the report of the Departmental Committee." We remember that a hope to this effect was expressed some months—or was it years?—since, but as the Local Government Board have taken upon themselves to advise the Ecclesall Guardians to continue a particular arrangement with respect to responsibility for administration for a time, it may, perhaps, be assumed that action is at last really contemplated. We note the words "certain of the matters" in the letter from Whitehall, from which it may be concluded that it is only intended to adopt a portion of the recommendations of the Departmental Committee. If a wise selection be made, the long delay in the issue of the Order will be excusable.

### MELANCHOLY DEATH OF A MATRON.

We greatly regret to record the death, under the saddest possible circumstances, of Miss Susan Mary Adams, matron of Addenbrooke's Hospital, Cambridge. It was only in December last that Miss Adams was appointed to this responsible position. She had previously been assistant matron at the David Lewis Northern Hospital, Liverpool, and had discharged her duties to the entire satisfaction of the governing body. Lately, however, she suffered from sleeplessness, which may or may not have been due to the fact that she acutely felt the responsibility of her office. On Sunday morning she left the hospital on her cycle, and was subsequently seen walking in the direction of Westwick Wood, seven miles from Cambridge. Two hours later she was discovered reclining against a tree, and upon examination life was found to be extinct. An inquest, of which we give a full report, was held on Monday, and medical evidence showed that death was due to poisoning by prussic acid. The jury found that the deceased took her life while temporarily insane. The verdict, we think, was the only one possible under the circumstances. At the David Lewis Northern Hospital, where Miss Adams was much esteemed, the event has created a very painful sensation.

### THE POSITION AT PLYMOUTH UNION INFIRMARY.

The latest development of the friction at Plymouth Union Infirmary is the resignation of an assistant nurse who has been in the service of the guardians for more than two years and a half. She has since, however, been induced to withdraw her resignation. The Local Government Board, not satisfied with the details furnished by the hospital committee respecting their relations with the superintendent nurse, have asked for more information, and it has been supplied. We share the desire of the hospital committee that the Local Government Board should take speedy action to bring about an alteration of the existing condition of affairs, but the latter are bound to know all that can be known before they can attempt to deal with the situation. At present the position at the Infirmary must be unpleasant all round.

### GUY'S TRAINED NURSES' INSTITUTION.

The report for 1904 of Guy's Hospital Trained Nurses' Institution shows that the twentieth year of its existence constitutes a record both as regards the income earned and the number of patients nursed. With respect to the former, the fees received for nurses' services amounted to £434 11s. 6d. in excess of the amount for 1903; and the assets exceeded the liabilities by £2,118 8s. 3d. The number of applications for nurses was 1,149, the number of persons nursed being 1,050, which, contrasted with 1,145 applications and 1,019 patients in 1903, indicates a larger proportion of work done. Another feature of the report is that 50 nurses shared in the bonus of £2,114, which was apportioned at the rate of £6 per share and invested in the Royal National Pension Fund for Nurses for their future provision. In all, the payments to the Pension Fund on behalf of nurses were £2,941 0s. 4d. Of the nurses who severed their connection with the institution last year, five returned to Guy's Hospital to take up positions as sisters; two became sisters in other English hospitals; one was appointed sister at Colombo Hospital; two went to private nursing institutions on the Continent; one became a Queen's nurse; 13 went into private nursing work on their own account; three returned home, two married, and one died.

### THE TORREY-ALEXANDER MISSION AND THE TEMPERANCE HOSPITAL.

On Thursday afternoon last week, Mr. Alexander, the American revivalist, and his wife visited the London Temperance Hospital. They were accompanied by Mr. Richard Cadbury and Miss Cadbury, and were met in the hall by the Matron, Miss Richardson, Mr. Vesey Strong, Chairman of the Board of Management, Dr. Robinson Souttar, Mrs. Strong, and several other ladies. Tea having been served in the Nurses' Home, which was of peculiar interest to the party owing to the fact that it was erected in 1887 at the cost of Mr. and Mrs. George Cadbury, the wards were inspected, and Mr. and Mrs. Alexander—whose sister was formerly a nurse at the London Temperance Hospital—stopped by the bedsides and spoke to the patients. In the women's medical block Mr. Alexander sang, accompanied on the piano by his wife, but in the men's ward some one with great energy called for the Glory Song. The request was at once complied with, the patients being invited to join in the chorus. A hymn set to an old plaintive melody produced a remarkable effect. Poor old "daddies" sobbed, young uncouth lads drew the sleeves of their red flannel jackets across their eyes, and tried to choke back the tears as the plaintive air flooded the ward. The silence which followed after told its own tale of appreciation.

### NURSES' NOTICES.

There should be no doubt whatever as to the date on which the notice of a nurse resigning her appointment takes effect. At the Newhaven Union Infirmary, however, a conflict of opinion has arisen on this point. The guardians passed a resolution to the effect that resignations date from the day they come before them at their ordinary meetings. But a nurse who recently sent in her resignation insisted upon leaving on the last day of the month from the time she gave notice, and nine days before it expired in accordance with the resolution of the guardians. In these circumstances, they have kept back her cheque for salary pending the decision of the Local Government Board. We apprehend that if the arrangement with the nurse was merely that she should give a month's notice, the Local Government Board will advise that the cheque should be at once forwarded to her. If the guardians require five or six weeks' notice, it is open to them to make such a stipulation the condition of an engagement, but a month's notice obviously ceases to be a month's notice if a number of days, according to the pleasure of the recipient, or recipients, are added.

### LIVERPOOL AND THE QUEEN'S NURSES.

In submitting the report of the Liverpool Queen Victoria District Nursing Association to the annual meeting, the chairman said that they closed the year 1904 with a deficit of over £2,000. An appeal had, however, been made by the Council to the citizens of Liverpool, with the result that the greater portion of that sum had now been subscribed. We think that the Liverpool people deserve commendation for the prompt manner in which they have responded to the appeal, but we agree with the Lord Mayor that greater regular support ought to be given to the organisation. During last year no fewer than 7,496 separate cases were attended by the nurses, and nearly 200,000 visits paid. In addition to this unique record, the nurses visiting the elementary schools for the purpose of attending to the minor ailments of the children made 46,695 dressings. An institution of this character in a city of such great importance and vast wealth as Liverpool should have at its back a sufficient amount in yearly subscriptions to enable it to make extensions without the fear of a deficit.

### GREENWICH GUARDIANS AND NURSES.

The Greenwich Guardians met the nursing staff of the Workhouse Infirmary face to face at their last meeting, and heard the objections of the nurses, through their spokeswoman, to some of the new regulations. The matron also was present to answer them. The result of the hearing was that the night nurses who objected to being compelled to return from their monthly leave at 1 p.m. when they had only to go on duty at 7 p.m., had their monthly leave fixed to extend from 7 a.m. to 5 p.m. on the following day. But in the case of both night and day nurses, who generally objected to a regulation that night and day duty should be taken alternately, it was decided that, as in the opinion of the clerk, the contracts could not be varied, and many of them had accepted their engagement specifically for night or day duty, the old conditions must stand. The night nurses, who are on duty every night, were also granted, as they asked, a half-day leave each week. If the nurses did not get all they wanted, they acknowledge the kindly spirit in which the Guardians endeavoured to meet their views.

### SENSATIONAL AFFAIR AT A LANCASHIRE HOSPITAL.

A very distressing occurrence took place on Monday at the Bootle Corporation Infectious Hospital, Linacre. A little before half-past three in the afternoon, the matron, going into the private room of Nurse Mary Edith Derham, found her lying on the bed suffering from the effects of carbolic acid poisoning. Medical aid was quickly at hand, but the nurse died shortly afterwards. She was about 27 years of age, and, though she had been at the hospital for only a month, was a favourite both with the nurses and the patients. Inquiries are being made into the matter, which so far remains a mystery. Miss Derham was the eldest daughter of the Chief Constable of Blackpool and had been a probationer in Derby.

### CLAYTON HOSPITAL, WAKEFIELD.

Satisfactory results were obtained at the annual examination of the probationers of Clayton Hospital, Wakefield, in elementary anatomy and physiology and medical and surgical nursing. Twelve probationers entered, six passed exceedingly well and five got through. The examiner's prize was awarded to Miss A. P. Gowland, who obtained full marks.

### THE NEW MATRON OF THE NEW HOSPITAL FOR WOMEN.

The election of a successor to Miss Cox Davies, formerly matron of the New Hospital for Women, Euston Road, and now matron of the Royal Free Hospital, took place on Thursday last week. There were several candidates, and the choice of the governors fell upon Miss K. Barling. Miss Barling was trained at St. Bartholomew's Hospital, and subsequently held in that institution the important post of night superintendent.

### SOCIAL MEETINGS AND STATE REGISTRATION.

A Social Meeting for Nurses and those interested in their work, now a well-established feature in the Somerset nursing world, was recently held at Kingston Grange, Taunton. Two circumstances combined to give the day a more than ordinary interest—viz. the announcement that a fête for nurses from all parts of the county would be held on May 30th at Moreton, by the invitation of Mrs. Barrett, and an address on the State Registration of Nurses, given by Miss Amy Hughes, who was introduced in a few warm words of welcome by the Hon. Mrs. Stanley. Miss Hughes spoke as an advocate of registration, but she recognised the point of view of its opponents, and her address was equally full of information to those in favour and those against the scheme, as well as to those who from various causes are still undecided which view to adopt. At the conclusion of her address Miss Hughes mentioned the scheme before the Board of Trade, which provided for a voluntary independent examination, and which could be started at once without an Act of Parliament. She considered that it might meet a need until State Registration could be obtained, as the London Obstetrical Society had done before the passing of the Midwives Act. A vote of thanks to Miss Hughes was carried with enthusiasm. A similar meeting was held the next day at the District Nurses' Home, Bristol, by permission of Miss Lloyd. Lady Symes occupied the chair. A resolution was passed *nem. con.*, "That this meeting considers that some system of State registration would be of advantage to the nursing profession and to the public."

### EXAMINATION AT KINGSTON UNION INFIRMARY.

The matron of Kingston Union Infirmary is to be congratulated upon the result of the second examination of probationers. Seven nurses were examined by Mr. Cantlie, of Charing Cross Hospital, who reports that the maximum number of marks obtainable was 100, and the percentages obtained 94, 90, 85, 78, 74, 66, and 60. Mr. Cantlie stated that the knowledge displayed in all branches was of a high order. At the last meeting of the Kingston Guardians special attention was drawn to the report, and much satisfaction was expressed by several members of the Board that they had nurses who entered so thoroughly into their work, and officers who took so much interest in training them.

### FOUR NEW NURSING ORGANISATIONS.

The continued development of the nursing movement is illustrated by the formation in a single week of four new organisations in different parts of the country. In Staffordshire the money is being rapidly collected for the establishment of a nurse for Norton-in-the-Moors. At Bishop's Castle it has been decided to form an association for that town, and to include the Salop portion of the parish of Lydham, with the Mayor as president. Thanks mainly to the vigorous efforts of Mr. and Mrs. Leng, newcomers in the district from Sheffield, who have lately taken up their residence at Welford Park, an addition is to be made to Berkshire organisations. A large committee has been formed, a series of rules adopted, and a cottage has been provided for the nurse by Colonel G. B. Archer-Houblon, who is to carry on work in the parishes of Welford and Boxford. Ottery St. Mary, in Devonshire, is also to have a district nurse.

### SHORT ITEMS.

The Chard Guardians, who had decided to pay fees for the registration of midwives, received two applications, but have refused to accede to them in consequence of an intimation from the Local Government Boai d that they do not possess the legal powers to carry out their intention.—We are asked to state that the fire at the Nurses' Hostel, to which we referred last week, occurred at 2.30 a.m., and not in the afternoon.

### The Nursing Outlook.

"From magnanimity, all fear above;
From nobler recompense, above applause,
Which owes to man's short outlook all its charm."

### THE DISTRICT NURSE AND HOSPITAL REFORM.

PATIENTS, practitioners and the public generally owe much to the trained nurse. The nursing world now offers so varied and wide a field for women workers that it enables every certificated nurse to choose for herself whether she will make as much money as possible in private nursing, or less money with less responsibility in hospital work, or possibly less money still and possess a wider field of individual influence for good as a district nurse in a large city? The district nurse of the latter type deserves well of all classes of the community. The advantages attaching to her work with the poor in great cities are immeasurably great and ever extending. She speedily introduces neatness, order, method, sanitation and comfort into the humblest dwellings. To be successful she must exhibit three main qualities, *i.e.* intelligent obedience to the doctor's orders, a love for the work, and great common sense. It is not easy to give entire satisfaction to one master, but the district nurse has to please many, for each of her cases may be under the care of a different practitioner. Very soon, as a successful district nurse, she obtains the confidence of the doctors; she proves an enormous help to patients and practitioners alike; and the doctors with her assistance find it practicable to undertake operations, and even to carry out courses of treatment in the houses of the poor, which they never could attempt without the assistance of the district nurse. It follows that the greater the success of the district nurse the greater is apt to be the volume of the work which she has each day to undertake.

To the members of a poor family in a crowded city, an efficient district nurse, frequently proves the angel in the house. Many nurses are, at first, inclined to think, that chronic cases are very tedious and uninteresting, yet untold misery is entailed in a small household by a case of cancer, for example, when no nursing worthy of the name is obtainable. In such chronic cases the nurse, by her knowledge and skill, can not only render the life of the sufferer relatively happy, but she can maintain a purity and sweetness in the atmosphere, rendering such a case almost, if not quite, unobjectionable, where, without her aid and attention, the lives of the healthy members of the family might, and often do become, a burden almost too grievous to be borne. In a large number of medical cases, too, a district nurse is able to render essential services to doctors and patients by the skilful administration of medicine, the preparation of baths, and in many other ways. In obstetric cases, also, the trained nurse is invaluable. She diminishes suffering, helps to preserve life, makes the work of the general practitioner infinitely less vexatious, and much more liveable, than can readily be understood by those who have no experience of such cases in poor districts. Small wonder if the life of many a district nurse is made happy as the day is long, for the love which she gives out to others in the course of her work may be returned to her a hundredfold.

A district nurse, too, in large cities, might be made to serve a material purpose in the economy of each hospital. At present London has far too few district nursing associations to adequately grapple with the multitude of cases which require such services in the homes of the poor. Yet the poor in a great city have a right to the attendance of the district nurse whenever there is illness in the home, and it should be the pride, as it is the privilege, of the relatively well-to-do, to supply the means to secure an adequate number of district nurses in each of the poorer districts. It was estimated, at the meeting of the South London District Nursing Association on Saturday, that about £20,000 is expended through district associations in London each year. Another £50,000 a year is required to extend the system sufficiently to meet the full claims of the poor within the metropolitan area.

At the present time it is generally admitted that the number of out-patients attended by the London hospitals is out of all proportion to the necessities and needs of the population. If every poor family could command the services of a district nurse the demands upon the out-patients department must largely diminish, for the poor find the loss of time involved in hospital attendance, a very grievous burden in not a few cases, and they would gladly avail themselves of a plan, which would save them this expenditure of time, and enable them, with the aid of the dispensaries, to have a doctor and a nurse in attendance, on suitable cases, at their own homes. Then, too, the almoners and inquiry officers of the hospitals could avail themselves of the services of the district nurse, and local medical practitioners, whereby they could ensure that all cases of abuse, so far as hospital attendance is concerned, should be at length effectually prevented. We hope that this aspect of district nursing, and of the usefulness and advantages of a district nurse, may soon be recognised by the hospitals, the public, the profession, and those generous donors by whose liberality our medical charities are at present maintained. If it is the cost of hospital work must be diminished, the abuse of hospitals may speedily be reduced to inconsiderable proportions, and the comfort and well-being of the poor in cities cannot fail to be promoted, to an extent, it would be difficult, perhaps, to exaggerate.

### Lectures upon the Nursing of Infectious Diseases.

By F. J. Woollacott, M.A., M.D., B.S.Oxon, D.P.H., Senior Assistant Medical Officer, Park Hospital, Metropolitan Asylums Board, Hither Green.

### LECTURE XVIII.—GENERAL MANAGEMENT OF ENTERIC FEVER.

There are few diseases in which nursing is of such supreme importance as in enteric fever, and, whether the attack be mild or severe, great care and watchfulness are necessary throughout. The same general principles should be followed as in the other infectious diseases. The sick-room should be kept at an even temperature of from 60° to 65° F., and plenty of fresh air should be admitted. The patient should wear a thin nightshirt, opening right down the back, and fastened by tapes. No other garment is necessary. The bedclothes covering him should be light, and consist of a sheet and a thin quilt only, blankets being undesirable during the febrile stage. There need be no fear that he will catch cold, and the absence of heavy clothing will tend to reduce his temperature. In the convalescent stage the clothing should be increased, and a vest and one or more blankets may be added with advantage. In the last lecture we saw that a striking feature of the disease was the exhaustion that so frequently attended it, the heart especially being seriously weakened. One of the most important objects of treatment, therefore, must be to economise the patient's strength as much as possible. He must be kept lying down in bed, and never, under any considerations, allowed to sit up. It should be impressed upon him that he must lie still, and ask for anything he may want, and not attempt to get it himself. Many patients are fond of half raising themselves and reaching over in order to get something from the locker or table by the bedside. This must not be allowed, and a tactful nurse will easily obtain obedience in this and other things without any suspicion of harshness. Sleep should be encouraged, and for this purpose the room or ward should be kept quiet. During the height of the fever the patient may be reduced to a state of complete helplessness, with all his faculties, both of body and mind, dulled and disordered. Under these circumstances he will be absolutely dependent on the nurse, and she must endeavour to anticipate his wants, so that he may not suffer from his inability to make them known. His position in bed should from time to time be changed, he should be kept scrupulously clean, fed at regular intervals, and a watch kept on the action of the bowels and the bladder.

*Toilet of the Patient.*—The whole body should be washed night and morning; soiled bedclothes should always be removed as soon as possible. As in other wasting diseases, in which the patient is kept constantly lying down, the continuous pressure of the bed is apt to irritate and inflame the parts that come in contact with it, and so give rise to *bed-sores.* The possibility of this should never be forgotten, and preventive measures should be begun from the very first day. There are two precautions by which the injurious effects of pressure may be minimised; in the first place, by using a soft, elastic mattress, of such a material as horse-hair, and keeping the bedclothes smooth and free from creases, and, secondly, by occasionally changing the patient's position, so that there is never pressure on the same part for any length of time. In addition we must take measures to keep the skin clean, and to improve its nutrition and resisting power. It is of great importance that it should be kept perfectly dry, and if there be incontinence of urine and fæces it is a good plan to protect the surface by some greasy application, such as lanoline or zinc ointment. In all cases the parts should be thoroughly cleansed with soap and water and then rubbed for a few minutes, two or three times a day, with weak spirit, and afterwards well dusted with zinc powder or starch. If the skin becomes tender, or begins to look red, water-pillows or a water bed are very useful, and white of egg or flexible collodion may be painted over the inflamed part. By strict attention to these precautions bed-sores may nearly always be prevented, but if, unfortunately, one should appear it must be reported on the next visit of the doctor.

The mouth and nose need constant care, and in severe cases should be attended to at least every four hours. The dried secretion should be removed from the nose, or, better still, prevented from collecting, and sordes should never be allowed to form on the teeth or lips. The mouth should be gently swabbed out at frequent intervals and, as far as possible, kept moist. This is particularly important after a meal, in order to remove any particles of food that have not been swallowed, and, if well enough, the patient should be taught to rinse it out himself. To keep the teeth clean a soft brush should be used. If cracks appear on the tongue and lips they may be painted with glycerine of borax or some similar preparation. Occasionally, especially if the mouth and throat are not regularly cleaned, inflammatory changes may be set up, which in time may extend to the ear, causing pain and discharge, or to the larynx, causing croup and more or less obstruction to the breathing.

*Diet.*—The diet of an enteric fever patient is of great importance for two reasons. In the first place, there is often much emaciation and exhaustion, and therefore the food must be as nourishing and as plentiful as possible, and in the second place, the intestines are inflamed and ulcerated, and therefore it must be easy of digestion, and of such a nature as not to set up still further irritation. The most usual plan is to give only liquids during the febrile stage, and for some few days afterwards. Milk is more generally useful than anything else, and five ounces of it may be given every two or three hours, so that two or three pints altogether are taken in the twenty-four hours. If the patient is sleeping he should not, as a rule, be awoke; but this is a point on which the medical attendant should be consulted. The milk may be sweetened or flavoured with tea or coffee, or otherwise, or diluted with lime or barley water. It is the common practice to allow an occasional meal of beef-tea or some other broth, and the change is often much appreciated by the patient, but it should be remembered that they contain very little nourishment, and are apt to increase the diarrhœa, although their stimulating effect may be useful. The patient may be allowed to drink freely water, toast water, or lemon-ade, carefully strained to remove all pips and solid particles, and during the twenty-four hours some five or six pints of liquid in all may be taken. A few days after the temperature has become normal the diet is gradually increased. At first only soft, pulpy foods are given, and many days, or even weeks, may elapse before the ordinary diet can be safely resumed.

The above account is only to be taken as a very brief outline of the ordinary routine practice in enteric fever. Many other articles of food such as whey, albumen water, jellies, cocoa, beaten up eggs, custards, etc., are frequently given in the acute stage of the disease, and in general it is advisable to introduce as much variety as possible, for a pure milk diet soon becomes monotonous and distasteful. Of recent years there has been a tendency to make still further modifications, and many medical men now allow solid food in the less serious cases when they consider the condition of the patient suitable. The advantages or otherwise of this course need not be discussed here, for the sole responsibility of ordering the diet must always rest with the doctor. It is the equally important duty of the nurse to see that the food ordered is properly prepared, that the patient is able to take it, and that he gets nothing whatever else. Alcoholic stimulants are frequently prescribed. They should always be given in measured doses and at stated intervals, like any other medicine.

*Reduction of Temperature.*—The wasting and exhaustion that attend enteric fever are largely due to the continuous high temperature, and it is therefore necessary to take measures for controlling and reducing it. This is most readily effected by the application of cold, in some form or other, to the surface of the patient's body. It is the custom to take the temperature every three or four hours and whenever it reaches a certain point, for example, 103° F., to take steps to bring it down. The methods in common use are too numerous and too well known to need any detailed description here. Sponging or packing, ice-cradling, or the application to the abdomen and lower ribs of large compresses wrung out of cold or ice water and frequently changed, may all be serviceable. A useful plan, and one which has the merit of being easily carried out with but little disturbance to the patient, is to employ the continuous pack, in which the patient may remain constantly for many hours or even days. A sheet, wrung out of tepid water, is wrapped round him and kept moist by sprinkling water upon it from time to time, so that it need not be changed unless it becomes soiled. Over the patient, thus wrapped up, a large cradle is to be placed and covered by a light quilt; and hot bottles should be put to the feet. One or other of these methods will be found satisfactory in most cases. They may usually be relied upon to effect a slight but definite fall of temperature with consequent amelioration in the patient's general condition. He will be soothed and quieted and may fall into a refreshing sleep. Unfortunately all these effects are very transient, and the treatment needs to be frequently repeated.

In some severe cases very little, if any, improvement results, and the temperature remains practically unaltered. In these cases the cold or tepid bath treatment may prove invaluable. Though comparatively little used in this country, it is carried out as a matter of routine in America and some parts of the Continent. The bath, filled with water at a temperature of about 70° F., is brought to the bedside, the patient is stripped, lifted on to a stretcher, to which a head-rest is fitted, and lowered bodily into the water. The head should be wrapped in a towel wrung out of cold water and the face frequently sponged. The duration of the bath is usually from ten to fifteen minutes, and during the whole of the time the limbs and chest must be well rubbed. The patient should then be lifted out, still on the stretcher, allowed to drain for a short time, and then put back to bed, wrapped in a dry sheet and loosely covered with a blanket. Hot bottles should be put to the feet, and if necessary some stimulant given. As might be expected the process is not a pleasant one. The patient often shivers violently and shows other signs of discomfort, but the advantages are undoubted. There is usually a considerable reduction of temperature, the delirium and tremor may cease for a time and sound sleep follow. The pulse improves, and the mouth and tongue tend to become moist. A useful modification of this method is to give the bath at a somewhat higher temperature, and if tepid water, from 75° F. to 85° F., or even more, be used, nearly as much benefit will result, and much of the discomfort will be avoided. The higher temperature is always advisable in the case of women and children. Another plan is to start the bath with water at about 90° F. and to cool it gradually by means of ice.

The continuous bath is occasionally employed. The temperature of the water is from 90° F. to 95° F., and the patient is kept in, if necessary, for several days. He must, of course, be carefully watched the whole time, and whenever the water is fouled with discharges it must be changed.

### Appointments.

[No charge is made for announcements under this head, and we are always glad to receive, and publish, appointments. The information, to insure accuracy, should be sent from the nurses themselves, and we cannot undertake to correct official announcements which may happen to be inaccurate. It is essential that in all cases the school of training should be given.]

Bradford Children's Hospital.—Miss Evelyn Mansel has been appointed matron. She was trained at Charing Cross and Guy's Hospitals, and has since been Queen's nurse at Southampton; sister at the Women's Hospital, Soho Square, London; nurse-matron at the Cottage Hospital, Easingwold, and at the Cottage Hospital, Ashby-de-la-Zouch; assistant matron at the Royal Hospital for Sick Children, Edinburgh; and matron at the Memorial Hospital, Kendal.

Brighton Workhouse Infirmary.—Miss Emily Florence Chorley has been appointed staff nurse. She was trained at Bath Infirmary.

Carshalton Cottage Hospital.—Miss Rose Battiscombe Mustard has been appointed nurse-matron. She was trained at Croydon General Hospital, where she has since been night sister.

Corbett General Hospital, Stourbridge.—Miss Emily Holland has been appointed staff nurse. She was trained at Crumpsall Infirmary, Manchester. She holds the certificate of the Central Midwives Board.

Hospital for Epilepsy and Paralysis, London.—Miss Gertrude H. Bean has been appointed sister. She was trained at Chesterfield and North Derbyshire Hospital and Dispensary. She has since been staff and charge nurse at the City of London Hospital for Diseases of the Chest.

Kingswinford Isolation Hospital.—Miss A. E. Gillespie has been appointed matron. She was trained at Fir Vale Infirmary, Sheffield, and has since been charge nurse at Coventry City Hospital; head nurse at Cannock Union Infirmary; nurse-matron at Hinckley Isolation Hospital; and head nurse at Ludlow Union Infirmary. She has also done two years' private nursing.

Luton Children's Sick and Convalescent Home, Luton.—Miss Jane Phelps has been appointed matron. She was trained at the Clayton Hospital, Wakefield, where she was appointed sister and night superintendent. She has since been assistant matron at the Prudhoe Memorial Convalescent Home, Whitley Bay, Newcastle. She has also done private nursing at Wolverhampton.

Monmouth Union Infirmary.—Miss L. Freeman has been appointed charge nurse. She was trained at Gloucester Union Infirmary, where she was afterwards staff nurse. She has since been nurse at the Garrison Women's Hospital, Woolwich.

Paddington Green Children's Hospital.—Miss E. Sheriff MacGregor has been appointed matron. She was trained at Westminster Hospital.

Royal Ear Hospital, Soho, London.—Miss B. Chamberlain has been appointed sister-matron. She was trained at St. Thomas's Hospital, London, and has since been a Queen's District Nurse. She has also been junior and senior assistant to the lady superintendent of the Nurses' Co-operation, London.

Ulverstone Hospital.—Miss M. L. Leng has been appointed matron. She was trained at the North Riding Infirmary, Middlesbrough, and has since been private nurse at the Sunderland Nursing Institute, temporary staff nurse at Kendal Hospital, and for the last three and a half years district nurse for the parish of Old Hill.

### South London District Nursing Association.

### THE USEFULNESS AND VALUE OF DISTRICT NURSES.

The annual meeting of the South London District Nursing Association was held at the Nurses' Home, Taybridge Road, Lavender Hill, on Saturday last (April 1st), Canon Erskine Clarke presiding.

In opening the proceedings the chairman referred to the fact that the volume of sickness which had to be dealt with by the association was continually increasing, owing to the great increase in the population. The ladies who took up the work acquitted themselves with enthusiasm of the duties laid upon them; they took with them into the homes of the poor the elements of sanitary conditions and instructed the relatives how to help the sick. It was something to be profoundly thankful for that such good work was going on and prospering.

Mr. W. H. Dickinson, the honorary secretary, moved the adoption of the report for the past year, which states that the visits paid during 1904 amounted to 41,268, or to 41,400 including 132 visits paid to persons it was not found necessary to attend again. The Board School attendances numbered 13,582 minor dressings, etc., of the children therein inspected. The number of new cases attended amount to 2,499. Of these 1,055 have been sent in by doctors and 107 by hospitals; 368 by ministers of religion or persons acting for them; 405 by patients' friends; 498 by teachers in schools; 26 by the Charity Organisation and other friends; 22 by the Invalid Children's Aid Society; and 18 by Poor Law authorities. Mr. Dickinson pointed out that the nurses had that year paid more visits than ever before, and more children had also been attended to. One of their friends had noticed that they had had to draw on their balance to the extent of £40, and he offered to make up half that deficit if the other £20 could be obtained in special donations before Easter.

Sir Henry Burdett said he felt it to be a great privilege to speak on behalf of that Association, especially when he looked at the list of names on their Council, many of them among the busiest men in London, and some of whom, like Mr. W. H. Dickinson, had given 21 years' voluntary service to that institution. But the work was one which everyone should do something to help. It was no use simply expressing gratitude at the beneficent results of the labour of others: that would not help man or woman on the planes of heaven. It was a work vitally important to the comfort, the lives, and the health of the children and of the poor of the district, and every resident in the district had an undoubted responsibility in the matter. Everyone ought to ask himself or herself what they were doing, and if they could do nothing else they might at least see the lady superintendent and get her to allow them to go round with one of the nurses; and if that did not convert them to the conviction that it would be a privilege to take part in the work it would at least bring them to a knowledge of the value of what was being done.

### Hospital and Private Nurses.

He had read their report with great interest because he had some knowledge of what the day's work meant. As they knew, he had for many years—for some fourteen consecutive years—lived in various hospitals, and in that way had acquired an intimate personal knowledge of all that appertained to the care of the sick in an institution. He did not know anything more trying than attendance on the sick there except, perhaps, the anxieties and responsibilities that devolved upon the district nurse. The nurse had a very hard time; she had grave responsibilities, and her remuneration was by no means excessive. To-day, when there were probably quite 30,000 trained certificated nurses, the state of things was very different from what it was twenty years ago when that association started and there were probably about 8,000 trained nurses in these islands. A nurse, when she was trained, could decide whether she would look on her profession as a matter of business or whether she would make love the first principle in her work. If a nurse selected private nursing she was able to earn in a good year from £100 to £150. That, in itself, though not a large sum, was sufficient for her needs, and enabled her by strict economy to put by something and so to save some £500 within the period of her working life of from 15 to 20 years. But a hospital nurse, though she was hard at work all day long, being employed in some hospitals for far longer hours than she ought to be, was paid very much less, the salaries of hospital nurses generally averaging some £30 a year.

### Hospitals and Nurses' Pensions.

On the other hand, hospital nurses could look on their employment as safe; they had a home, food, and pocket-money; but he thought it very necessary that each institution should take care that a large proportion of the profits they made out of private nursing should be set aside for pensions or retiring allowances for the nurses. (Applause.) At Guy's Hospital the system they had there had just resulted in giving one nurse £70 as a retiring pension, and he had been going into the] figures of that scheme, and he was glad to find that the prospect was that few if any, nurses would get less than £60 a year pension. That fact proved that some institutions supplying nurses to the public which made no pension provision for their staff must be making large profits. And as these institutions were most of them managed by men of character and goodwill, he thought they would do well to look into the question and decide at once if in London at least they could not follow the lines adopted at Guy's and so be just to their nurses.

### The Work of the District Nurse.

But, apart from the private nurses and those who worked in hospitals and devoted their lives to the care of the sick, there was the largest class of nurses, those who became district nurses and laboured among the poor in their own homes. One feature in the system was very notable, the interest which nursing possessed for every woman. At the very commencement of the Metropolitan Nursing Association it was the idea of Mrs. Dacre Craven that every nurse employed should be a gentlewoman, and so be in a position to give the best possible help to the sick poor. That idea was received at the time with much criticism. It was maintained that they would never get ladies to devote their lives to such menial work as devolved upon district nurses; that it was a mistake to suppose that ladies would undertake the work, and that if they did they would fail because they would break down under the strain. Putting aside the fact that no work could be menial which had for its object the helping of the sick who could not help themselves, and the increase of the comfort and health of the homestead, they had there in Battersea proof positive that Mrs. Dacre Craven's idea was a sound one. They had, in fact, been carrying it out successfully for 21 years. However much one might extol the life and mission of nurses in hospitals they certainly ought to give a proper meed of praise to those ladies who continued year after year their labour of love in the homes of the people. District nursing meant much to three classes—the poor, the doctors, and the nurses themselves. The nurse who first began to work in the homes of the people often found a state of things not only unknown to but unrealisable by most people. Not one in a hundred thousand had the smallest conception of the actual conditions in which many of the households of the poor were carried on to-day. He had inspected the homes of the poor in many large towns, and especially in London, and so had a personal knowledge of the state of things in which the poorest families had to exist. A district nurse, on her first visit to such a family, found very often disorder, discomfort, and frequently dirt and insanitation, very trying to face; yet they required their nurses to undertake to clean the rooms, purify the bedding, and make the place tidy and comfortable generally, in addition to her proper work in connection with the patient. He always felt that every district nurse must be a woman of character and common sense, inspired by the motive of love, and possessing intuition and tact, with great power not only to make the best of things for the patient, but always to give satisfaction to the doctor. It was not so very difficult for a clever woman to manage one man—(laughter)—but when there were many, and all doctors, it might prove less easy.

District Nurses and Hospital Reform.

This question of district nursing was intimately bound up with the life of London. They had had the hospital outpatient abuse difficulty going on for years. The time had come to face it resolutely, however, for the longer we delayed facing an evil the more it would grow and the more it would cost in the end. He believed that an extension of district nursing on the lines of that Association in every part of London would do more to help on and expedite a solution of the outpatient question than anything else. (Applause.) When we come to look at London, with its four or five million inhabitants, the difficulties in the way of reform seemed at first to be insuperable. The key to the solution was to get into touch with the homes of the people who used the hospitals, and in that way take the wisest step to effective reform. What was wanted was knowledge and the means to raise the self-respect of the people. If London were wise it would insist on such a development of district nursing as would cover every district with an Association similar to that one, so that district nurses could be employed in the homes of all the people needing them, with the result that directly the nurse became known and began her visits the comfort and health of each family and the work of each doctor would be immensely improved. With the coming of the nurse would come the awakening of better ideas, which would tend to make each householder join a dispensary and so secure medical attendance at home in case of need, which with the help of the nurse would prove even better in many cases than going into a hospital. Of course such an arrangement would take time. Looking at the needs of London, and estimating very roughly, he thought that what was required was something like £70,000 in all to be spent in district nursing. At present about £20,000 was being expended each year, so that another £50,000 was needed to supplement the work of the existing District Associations, for he was confident the results would then be infinitely better and quicker than could be hoped for from starting a number of new organisations. His object in mentioning this idea, then, was to bring it to the notice of some of the generous and wealthy people in that great city so that the poor might come by their rights.

The Work at Battersea.

What that institution in particular wanted to carry on its part of the work was a little more money, some £200 a year. Their work last year represented some 2,500 cases—a tenfold increase in 21 years; but the lamentable thing was that the subscriptions from the district had been reduced by one-tenth. That was a state of affairs which, if they could bring it home to all the people of Battersea who had brains and ideals, must soon be remedied. Let them consider the cost of the work—five shillings a case! and a total expenditure of £1,000. Everyone who gave £1 knew that he was providing for the relief of five poor people in their own homes in the day of sickness. Where could they get better value for their money? Seven nurses were employed, so that each had been attending, in addition to her old cases, some 360 new cases during 1904—a new case for every day in the year. The presence of the district nurse added to the welfare of the family as well as to that of the patient; it shed a ray of sunshine which gave new aspirations in life and new hope to existence in the homes of the poor. They might, therefore, regard the work of the district nurse as amongst the highest, noblest, and most useful callings open to women.

The Rev. Arthur Jephson, L.C.C., seconded the resolution, which was carried, after which Mr. Percy Thornton, M.P., moved the re-election of the President, Chairman, Hon. Secretary, and Council, which also was adopted, and a vote of thanks to the Chairman concluded the proceedings.

### Scottish Nurses and the Pension Fund.

### INTERVIEW WITH THE SECRETARY.

In view of the probability that some of our readers may not be aware of the advantages of belonging to the Royal National Pension Fund for Nurses, and of the possibility that some do not even know of its existence, I asked Mr. Louis H. M. Dick, the secretary, to see me at the offices in Finsbury Pavement in order to learn the result of his recent visit to Scotland, and to obtain any other information that might be useful. The Hospital has, of course, co-operated with the Fund from the time it was founded, but in spite of that, as Mr. Dick's first words show, there is still room for a good deal of illumination respecting its objects.

"In Scotland," he said, "I find that the Fund in many places is quite unknown, and that where it is known it is a mere name. As an instance of this, I may mention that one matron, answering an invitation to a meeting in Scotland, replied that she knew nothing about the Fund, and did not want to know anything. The incident, I think, indicates the lack of interest on the part of the Scottish nurses, as well as of the matron, who could express such an opinion without trying to induce her nurses to become less ignorant. In contrast to this, as a result of my visit to Glasgow and Edinburgh, I received sufficient invitations to speak at other institutions to detain me in the neighbourhood of the two cities for a week."

"Why did you go to Scotland?"

"The origin of my visit was to speak to the nurses at the Victoria Infirmary, Glasgow, in consequence of the infirmary having federated with the Pension Fund—the first Scottish hospital to take that step. By the courtesy of the House Committee this meeting, which was meant to be a purely institutional gathering, developed into one embracing all the nurses in Glasgow who cared to attend. Practically, there were present at the meeting representatives from almost every hospital and nursing institution in the city. The chair was taken by Dr. Clark, C.M.G., one of the surgeons of the

### SCOTTISH NURSES AND THE PENSION FUND—*Continued.*

Glasgow Royal Infirmary, supported by Miss Macfarlane, the matron, and Dr. Macgregor, the medical superintendent.

### The Influence of Matrons.

"Were there many matrons at the meeting?"

"A very large number. Your inquiry raises the question of the influence of matrons, and prompts me to mention an interview which I had with a matron here on Monday. She told me that for years she had not joined the Pension Fund because it was not necessary for her to do so, but that her family having suffered heavy pecuniary losses, she was compelled to make some sort of provision for herself. That happened a year ago, and since she joined she found that she had not the slightest difficulty in inducing her staff to join also. She then expressed her regret that the matron of her training-school had not impressed the value of the Fund upon her, and she said that, in her judgment, matrons do not realise the extent to which they can influence their staff. She added that many, doubtless, think it is a kind of interference with the liberty of the subject to touch upon any topic outside training and discipline, when, as a matter of fact, nurses would not resent a suggestion from a much older woman than themselves, that they should endeavour to manifest a little independence of spirit by making some provision for later years, however slight."

### Dr. Affleck on the Fund.

"You had also, I believe, a great meeting at Edinburgh?"

"It was held, by permission of the managers, in the theatre of the Royal Infirmary, and was an extremely interesting gathering. The chairman was Dr. Affleck. We were especially happy in having him, not merely because he has always been a warm advocate of the Fund, but also because he is personally a great favourite with the nurses. Moreover, he has an intimate knowledge of insurance matters, as he happens to be the medical adviser of one of the most prominent of Edinburgh Life offices. Dr. Affleck gave a short, but very weighty address, in which he strongly emphasised the urgent need of making some start on the road to thrift. There were several ways, he observed, of doing it—through an insurance office, through the Post Office, or through the Pension Fund. Having known the Pension Fund from its inception, and watched its progress with the closest attention, he declared that he was perfectly satisfied that the nurses could not do better for themselves than by becoming members of the Fund. He explained that so far as the head nurses of the Edinburgh Royal Infirmary are concerned, provision is made by the Infirmary scheme of superannuation, but, having pointed out that there can only be a certain number of head nurses, he concluded by warmly recommending the others to join the Fund. The superannuation scheme, by the way, is a most liberal one, though as Dr. Affleck says, its benefits are limited to the seniors.

"Miss Spencer, the matron, and all her assistant matrons were present, working very hard to make the meeting the success which it was. Colonel Warburton, C.S.I., medical superintendent, and Mr. W. S. Caw, the treasurer and secretary, were also in the audience, as well as Miss Guthrie-Wright, the honorary secretary of Queen Victoria's Jubilee Institute for Scotland, many matrons, and over 200 nurses."

"At what other institutions did you speak?"

"The Nurses' Club in Glasgow; the Stobhill Hospital, Glasgow, in response to a cordial invitation from Miss Wright, the matron, and the Royal Scottish Nursing Institution, 69 Queen Street, Edinburgh."

### The Prospects in Scotland.

"I should be glad to learn what is the general impression created upon your mind by the visit."

"It is most emphatically that there is a splendid opening for the Pension Fund in Scotland. This view is not founded alone on what the matrons have told me, but also on what has been said to me in personal conversation with a large number of hospital and private nurses. On the eve of my departure from London I received a letter from Miss Peter, superintendent of Queen Victoria's Jubilee Institute, which was of material assistance to me and which I read at the meetings. She wrote, 'I am much interested to hear that you are going to Scotland to explain the Pension Fund to the nurses there. I feel sure that "explainings" are much wanted, and that the Scotch nurses, when they fully understand the great advantages of such a Fund, will join in numbers. As you know, a great number of the Queen's nurses are members, and often say to me and write how very glad they are that they have joined. Then the Benevolent Fund. No other pension fund, I think, has such a helper by its side, and so many benefit by that, not only by having their premiums paid during illness, but are helped to get quickly well by having expenses paid to a convalescent home, etc. I trust that your visit will result in numbers joining.' My own experience is that those who are already members in Scotland are very enthusiastic and anxious to augment the list of members north of the Tweed."

"All this must be exceedingly encouraging to you?"

### The Report to Policy-Holders.

"It is, but the very satisfaction with the Fund has an effect which is prejudicial to it. For example, a report is sent to every member of the Fund. But a large number of nurses are so profoundly satisfied with its progress that they do not take the trouble to read the report. As you are aware, the report this year was accompanied by a letter containing a certain suggestion which, if acted upon, would probably assist to secure fresh members. I want those members who have not opened the envelope to do so, and peruse the letter. Or if they have mislaid it, and will let me know, another will be forwarded at once. My object is that in 1905 the number of new members shall quite eclipse the numbers who enrolled in 1904, which was our record year."

### General Progress.

"It might be useful if, at this point, you could say something about the general progress of the Fund."

"Each year since 1900 the proportion of recruits has been greater than the previous twelve months. As regards 1904 this is all the more striking because it was what is known as a bad year for nurses, owing to all-round depression. The inference is that many nurses realised for the first time, the possibility that they would not continue to have a regular succession of cases. I found this assumption on the fact that the average age of new admissions was considerably higher than it was during previous years, while the amount for which policies were taken out was lower. When a nurse is young, she does not think £15 or £20 a year at the age of 50 good enough to trouble about, but when a few more years have been added to her life, she comes to the Pension Fund with the money in her hand, so to speak, and says, 'This is all I can afford; do the best you can for me.'"

### No Politics.

"There is one thing," Mr. Dick continued, "I should particularly like to mention. I am constantly being asked my views on State Registration, and my very emphatic answer, which I repeat now, is that the Pension Fund has no politics; therefore, I, in my official capacity have none. We have amongst our policy-holders' representatives, ladies who hold views diametrically opposed on the subject. But they are all agreed upon one point—namely, that to make easy and adequate provision for her future, the nurse cannot do better than join the Pension Fund."

### Tragic Death of the Matron of Addenbrooke's Hospital, Cambridge.

INQUEST AND VERDICT.

An inquest on the body of Miss Susan Mary Adams, matron of Addenbrooke's Hospital, Cambridge, who was found dead at Westwick on Sunday morning, was held at the New Inn, Oakington, on Monday, by the County Coroner.

### Evidence of the Deceased's Brother-in-Law.

The first witness called was Mr. James Niven, a medical practitioner, of Manchester, brother-in-law of the deceased, who gave evidence of identification. Miss Adams, he believed, was about 31 years of age. She had written to witness's wife regularly during the time she had been matron of Addenbrooke's Hospital. She had mentioned worries in connection with her administration of the hospital, and seemed somewhat depressed in consequence. He had never known her threaten to commit suicide. She always appeared to him eminently sane up to recently. In the various things she said in the letters he could now see that they indicated a considerable mental alteration. For instance, she mentioned that she had spoken to a member of the committee asking him whether it was not possible for her not to have to go before the House Committee. That seemed to him overstrained at the time, but he thought that it really was not a matter of great moment. He knew that she had said that all the members of the committee had been exceedingly kind to her and she had absolutely nothing to dread, and he now realised that such a degree of alarm as that over a matter which could not possibly give her any distress must have indicated some mental alteration. Then there were other matters and little worries connected with her own administration of the nurses which he would not have thought very much about had it not been for the change. She kept recurring to those matters; but, at the same time, neither he nor the family had attached very much importance to them.

### The Deceased at Westwick.

A porter in the employ of the Great Eastern Railway Company stated that he saw the deceased at the level crossing about 8 o'clock on Sunday morning. She walked across the line, and the signalman then informed him that she had put her bicycle over the hedge. Witness called the deceased back to ask her if she wished to take her bicycle over the crossing. She did so, and cycled off through Westwick. She was alone.

The wife of a farm labourer, of Westwick, deposed that about five minutes to eight on Sunday morning the deceased asked witness if she might leave her cycle at her house. Permission was given, and the deceased went in the direction of Cottenham. She said it was windy, and she was going to walk.

A milkman, of Westwick, stated that at about eight o'clock he saw the deceased on the Westwick Farm premises. She was walking towards the wood, where she was subsequently found.

The foreman at a farm, who found the body of the deceased, said he was walking in Westwick Wood about ten o'clock, and saw Miss Adams sitting against a tree. He went to her, could not see her breathe, and went away to give information to his master, with whom he returned. They concluded she was dead, and sent for a police constable. The body was removed to the New Inn.

### Evidence of the Matron's Assistant.

Miss Emma Spencer, matron's assistant at Addenbrooke's Hospital, said that she had been in constant communication with Miss Adams during the last three months.

The Coroner: Recently has she found her duties rather irksome?

Witness: I believe she has been tired.

And different to what she was when you first knew her?—Yes.

Was that about three months ago?—Yes.

Did she complain of anything?—No, except sleeplessness.

Did she express dislike to performing any part of her duties?—No.

Going before the committee or anything of that kind?—She seemed to feel the responsibility of it.

Did she ever threaten to commit suicide?—No.

Or say anything to that effect?—No, she never threatened anything of the kind.

Yesterday morning did she leave the hospital about six?—At a quarter to seven, I believe.

Did she say where she was going?—I did not see her yesterday morning.

Did she leave a note?—No, not that I know of.

How long had she suffered from sleeplessness?—I cannot say how long—the last two or three days. A few days before she told me she was sleeping better.

### The Medical Evidence.

Mr. Albert Julian Pell, chairman of the general committee of the hospital, stated that Miss Adams was appointed matron of the hospital in December last. She then gave her age as 31. She was selected out of 52 applicants, and came with most excellent testimonials. During the short time she had held the office of matron witness thought she had carried out her duties to the entire satisfaction of the whole of the committee. To his knowledge she had not been found fault with in the smallest particular. It had been mentioned by one of the witnesses that she felt somewhat nervous about appearing before the committee, That only entailed her being present for two or three minutes to produce her books which contained the record for the past week. The books were just examined and the matron went out. She had no cause to worry so far as he knew. He saw her that week, and she seemed just as usual.

Mr. F. E. Apthorpe Webb, surgeon, Cambridge, who made a post-mortem examination, said he found in the stomach traces of prussic acid. The pupils of the eyes were dilated. The cause of death, in his opinion, was poisoning by prussic acid. The reaction in the test for the poison was such as could not have been produced by the medicinal use of the poison.

Mr. C. H. Cox, medical practitioner, of Cottenham, who was called to see the deceased in the wood, described the position in which the body was found. In the deceased's jacket pocket he found some freshly-picked violets. He assisted Mr. Apthorpe Webb in making the post-mortem examination and agreed with his evidence.

Mr. William Joy Field, dispenser at Addenbrooke's Hospital, said that the poisons at the hospital were kept in a locked poison chest, to which the deceased would not have had access. Witness had never supplied her with poison. He thought that it was impossible for the deceased to have obtained prussic acid at the hospital unknown to him.

The jury returned a verdict of "Suicide during temporary insanity," and expressed sympathy with the relatives of the deceased.

### Nursing Lectures under the London County Council.

BY ONE OF THE LECTURERS.

In view of the fact that the subject of physical degeneration is arousing a growing interest in the homes and needs of the poor, it may be found instructive to see what the London County Council is doing for the spread of a knowledge of the simple rules of healthy living, of the prevention of disease, and of the manner in which children should be brought up. For some years free evening classes have been established in the London Board Schools, both for men and women, which are continued for twelve weeks, one class being held each week, provided an attendance of 16 members can be maintained. A syllabus is drawn up by the medical superintendent in charge of the department which includes both practical and theoretical instruction in the elements of sick nursing, hygiene, and first aid. The lectures are given by doctors or trained nurses with some assistance from a teacher who holds the London County Council certificate for the teacher's course arranged by them.

### New Classes.

They now propose, and have indeed started, some other classes for women and girls in which the principal object is to teach the best method of rearing young children. Those who have worked much among the poor well know the tremendous number of prejudices which will have to be fought before new and sane ideas can be instilled into their minds and be made a routine of daily practice. The idea that to wash a sick person is harmful, that night air is dangerous, that babies may be fed on the same food as their parents, that brandy is a panacea for every ill are beliefs deeply rooted and not easily eradicated. Being called upon to take a series of these classes I found it a very different thing to teaching a well-disciplined class of probationers. The members consisted of girls coming in from shops after work hours and married women willing to get some knowledge, presumably to help in their homes in time of sickness.

### The Need of Instruction.

I was wholly unprepared for the ignorance displayed. Thinking I had been as simple and elementary as possible when describing a state of active delirium, I was surprised to be asked "if the patient could help it." It proved easy to arouse attention and impress facts by the graphic description of a case illustrating the point under consideration—such description drawing exclamations of sympathy or of horror from the pupils in an amusing manner. Special stress is laid upon the prevention of the spread of infectious diseases; a full description of the early symptoms being given and of the various periods of invasion, isolation, and disinfection. The importance of ventilation is dwelt upon, and the rearing of infants and diseases of childhood are made of prime importance. The practical work includes bandaging, bed-making, poultice making, taking and charting of temperatures, etc. This part of the work appears to present the greatest difficulty for the women, not always attending regularly and not being at all expert with their fingers, would require many more lessons to make them proficient. This, however, proves the immense need of instruction, for it must often happen that some work of the kind falls to a mother's share, and that she should know how to do it is essential to the well-being of the family.

### The Practical Value.

The necessity of keeping so many children away from school to attend hospital for small ailments will be obviated when the women understand better what to do in slight cases and how to do it. For the men the lectures on first aid will often prove of direct benefit to themselves and others, and promotion in the police force depends now upon the gaining of a certificate for proficiency in this branch. That both men and women in all classes of life should have a very definite, if elementary, knowledge of the laws which govern health and prevent disease nobody will question, and the London County Council will do the country great service if it fulfils this purpose.

### The Foundations of Health.

There was a large attendance last week at the Institute of Hygiene, Devonshire Street, the occasion being a lecture by Dr. W. G. McDowell on "The Foundations of Health—First Year of Infancy."

After premising that he did not propose to enter into all the details of infant management during the first year of life, but only to refer to those points whereon ignorance prevailed among the people, Dr. McDowell dealt with some important matters of common belief which are at variance with fact and inimical to the health of the young infant. He referred to the craze for superfatting the milk upon which infants are fed, and quoted some records by Dr. Emmott Holt concerning cases in which, though the first results were satisfactory—the children growing plump and apparently healthy—they early developed obstinate constipation and finally suffered from convulsions. The dejections of these infants were examined and found to be masses of little else but pure fat, though the fat in the milk had only been increased from four to five or six per cent. These children recovered when the fat of the milk was reduced by skimming to half the normal percentage; but in how many cases, asked the lecturer, in the present state of popular knowledge, would the very opposite method be adopted, that is "the cream further increased and the child lost in consequence?"

Dr. McDowell then pointed out that the secretions of the pancreas and the liver, which in the adult are physiologically employed in dealing with fat for absorption, are not capable of fulfilling this function in early infancy; the consequence being that only a small proportion of the fat consumed is absorbed from the alimentary canal, the rest passing on—the only constituent of milk that is not used up—to give bulk to the dejections and assist the expulsion of effete material.

The lecturer inveighed against the reference of children's ailments to dentition, which he said was the cause of many preventable deaths, and seldom justified by fact. If the mother would think first of the stomach and feeding, of fresh air, of the changes going on in the nervous system, in the bones, and other parts, and of the possible ills of heredity, she would be more likely to get into touch with the real truth and seek the guidance of the medical attendant before it was too late. It is great folly to depend on the old delusive formula, "It is the teeth," until the condition becomes hopeless.

Finally, Dr. McDowell protested against the presence of preservative in milk intended for the use of infants. To give the unhygienic dairyman the privilege of substituting boric acid for cleanliness and thoroughness in his operations was a legislative iniquity against which no sufficient outcry had yet been raised. From the *American Journal of Medical Science* for September last he quoted the record of a series of experiments which went to show that from six to ten grains per diem of this preservative was sufficient to kill one cat out of six in 42 days, and to bring on kidney disease in the other five. Yet the dairyman might give it to our young infants, and in oft repeated doses, without our knowledge or consent.

### Prevention of Tuberculosis.

On Friday afternoon Dr. J. E. Squire gave a lecture, at 20 Hanover Square, on "Tuberculosis and its Prevention," for the benefit of health and district visitors and other workers among the poor. Most of those present were ladies, and there was a sprinkling of nurses in uniform.

Dr. Theodore Williams, who occupied the chair, said that the object of the lecture was to excite a tendency, not among the medical men and women of England, but among the lay members of the community, to do something to prevent this terrible scourge. Referring to Dr. Bodington as the introducer of the open-air treatment, the chairman recalled the history of the study of the disease during the last 20 years, and urged all present to take a share in helping the doctors to stamp it out.

Dr. Squire (physician to the Mount Vernon Hospital for Consumption and Diseases of the Chest) said that although the mortality from the disease was nearly 60,000 in England and Wales alone, the gradual improvement in the general hygienic conditions of the people had resulted in a steady fall in the annual death-rate from consumption and that with special precautions, based on a more complete knowledge of the determining cause of tuberculosis, a far greater improvement might be confidently anticipated. To gain the co-operation of the public, the people must be educated to recognise the necessity for, and to appreciate the reasons underlying, the prophylactic measures recommended, which were based on a knowledge of the causation of the disease, and of the conditions favouring its spread and rendering persons susceptible. Preventive measures must be directed towards (1) destroying the infecting germ given off by the sick; (2) preventing or counteracting the susceptibility of the individual. The two chief agents of infection being expectorated matter and the milk of tuberculous animals, destruction of germs before the expectoration became dried and pulverised, and boiling the milk, were the two chief means of effecting the desired end. By means of maps, the lecturer showed how the death-rate from consumption in London corresponded very closely with the overcrowded areas, where the deficiency of fresh air and sunlight, together with poverty and consequent lack of sufficient nourishing food, was a powerfully predisposing factor; while a chart showed that with the rise in wages and the cheapening of food during the last 40 years, the death-rate from consumption throughout the country had considerably declined. The lecturer concluded by giving some practical hints as to the care of the sick person's room, *e.g.*, the use of wet dusters, and the admission of the maximum of sunshine and fresh air, remarking that the rules of life comprised under the term "general hygiene" furnished the key to the prevention of a predisposition to the disease, which was not solely a matter for the medical profession, but required the intelligent co-operation of the public.

The chairman, in thanking Dr. Squire, said that they had listened to an exceptionally lucid account of what was in reality a very complex subject, and urged upon those present to carry out the advice given them in their work among the poor, adding that it was quite conceivable that, with improved conditions of life, the disease might be effectually eradicated.

### "The Hospital" Convalescent Fund.

The Hon. Secretary acknowledges with many thanks the receipt of 5s. from Miss A. B. Bailey and 5s. from the Travel Correspondent of The Hospital.

### Everybody's Opinion.

[Correspondence on all subjects is invited, but we cannot in any way be responsible for the opinions expressed by our correspondents. No communication can be entertained if the name and address of the correspondent are not given as a guarantee of good faith, but not necessarily for publication. All correspondents should write on one side of the paper only.]

### OVER-WORK IN A COUNTRY UNION INFIRMARY.

"H. W." writes: With all the modern improvements in science and the great improvements of our hospitals of to-day, it is remarkable how very far behind the times our country union infirmaries are. The women who take up nursing in these places are plucky and self-sacrificing, as the many difficulties they have to contend with and the thousand and one drawbacks and hindrances they have in their work are enough to take the heart out of anyone. As everyone knows, a nurse's life is always more or less hard, and the nurse in the country union infirmaries has the hardest life of any, because they are so dreadfully understaffed, consequently the nurses get overworked, and then their health has to suffer. I know of one where the head nurse looks absolutely worn out, yet she keeps on, and before the patients always has a smile and a kind word. Day after day she gives up her off-duty time, because the wards are full and there are lots of very bad cases with a very small staff, and she likes her nurses to get their time off if she cannot. Even this cannot always be managed. Such a state of things cannot be right. The nurse's health ought to be considered: she spends all her time with sick and dying people, and she should have her two hours completely off duty daily, in order that she may get right away from the atmosphere of sickness. The guardians would possibly plead that they have no money, but surely there is some way of finding the means, if not for building improvements, for supplying a full staff of nurses.

### A NOVEL PROPOSAL.

Trained Nurse writes:—It has come to my notice lately that several untrained and uncertificated nurses are going about taking situations and wearing nurses' uniform. They are imposing on the public, who, ignorant of the fact that these nurses have had no training, rely entirely upon them and place in them every confidence. Besides this, they take the practice away from us who are working on "our own," and who have been through a long course of training and hold certificates for efficiency. It is only through my having been a loser myself lately that I felt I must write to you on behalf of my fellow-workers who may be suffering from the same cause. Is this right? And if not, may a remedy be provided? I would suggest that each certificated nurse should have a medal with the initials C.N. (Certificated Nurse), to be worn on the right shoulder, conspicuous to all, and only purchasable by those who can produce proper certificates—not copies—a penalty being enforced on any one selling a medal to any nurse not provided with such certificate. May I suggest that if you think the plan feasible, that these medals should be purchased only from one firm. This fact might be inserted in the daily papers, and in that way the attention of the public would be secured.

### To Nurses.

We invite contributions from any of our readers, and shall be glad to pay for "Notes on News from the Nursing World," or for articles describing nursing experiences at home or abroad dealing with any nursing question from an original point of view according to length. The minimum payment is 5s. Contributions on topical subjects are specially welcome. Notices of appointments, letters, entertainments, presentations, and deaths are not paid for, but we are always glad to receive them. All rejected manuscripts are returned in due course, and all payments for manuscripts used are made as early as possible after the beginning of each quarter.

### The Metropolitan Asylums Board and the Staff at the Ringworm School.

At a meeting of the Metropolitan Asylums Board on Saturday last the Children's Committee reported that they had had under consideration a report from the dermatologist at the Bridge Ringworm School, Witham, Essex, and had conferred with him upon the question of obtaining better results at the school by increasing the skilled supervision. At present the arrangement was that each of the three charge nurses, with an assistant nurse, was responsible for the care and treatment of 50 children, and there was a sister over them who supervised their work under the matron. This arrangement had, however, been found in practice to be unworkable, in view of the impossibility of one person effectively supervising the treatment of 160 heads in three different centres. The committee therefore proposed to divide the school, for purposes of treatment, into two sections, with 80 children in each, and to place in each section a superintendent nurse, a charge nurse, and two assistant nurses. The salary of the superintendent nurse would be £42 per annum, rising by annual increments of £1 to £44 per annum, with board, lodging, washing, and uniform. The committee recommended accordingly, and their report was agreed to. The committee added that the present holder of the office of sister was in such a state of health that they did not think it advisable to retain her services, and it was decided, subject to the sanction of the Local Government Board, to grant her a gratuity of £40 as compensation for the loss of her office.

### Presentations.

Ardwick and Ancoats District Nurses' Home. — The medical practitioners with whom Miss Blower, late matron of the Ardwick and Ancoats District Nurses' Home, has been associated for the past nineteen years in the work among the sick poor of the district, have presented her with an illuminated address and cheque as a token of their esteem and high appreciation of her services to them in the care and skilful nursing of their patients. The presentation took place on Tuesday afternoon. Dr. E. Vipont Brown presided, and Dr. Saberton, on behalf of over sixty medical men, whose names the address bears, made the presentation.

### Where to Go.

The Dutch Gallery, Grafton Street.—Lovers of flowers will be well repaid by a visit to see Mr. James's beautiful collection of water-colours now on view at the Dutch Galleries. The exhibition is devoted entirely to floral studies, and these are of such striking merit as to render the collection practically unique. Mr. James's command over his subject is especially shown in his successful effects produced by white flowers on a white ground. It shows us white primulat treated thus with the same incisive character which is presens in the most brilliant of his groupings.

Brixton Theatre.—During the present week Mr. Louis Calvert's company are giving performances of Mr. Thomas Raceward's charming play, "Sunday." We think other readers besides those who live conveniently near Brixton may like to know that the company is going on tour throughout the country, and we can confidently recommend nurses to take an opportunity, if it occurs, to see "Sunday." It is well acted, and its bright and healthy tone is refreshing. The author is somewhat lenient to those who take the law into their own hands, but we do not think that our readers are in danger of emulating this characteristic.

### TRAVEL NOTES AND QUERIES.

By our Travel Correspondent.

The Orkneys (Stecton).—The steamers of the North of Scotland, Orkney, and Shetland Steam Navigation Company sail three times a week in summer from Leith Pier, Edinburgh, to Kirkwall or Stromness. Time occupied between Edinburgh and Kirkwall is 24 hours. I do not know the cost, but you will get all information by writing to the office of the company, 16 Waterloo Place, Edinburgh. As to apartments no doubt they are to be obtained, but you would probably be moving about from place to place, so that hotels would be more suitable. At Kirkwall go to Black's Temperance Hotel. At Stromness "The Commercial," in Victoria Street. If you go on to the Shetland Isles, at Lerwick go to Crutwell's Temperance Hotel. If you want more help let me know.

A Holday in the Tyrol (Robin).—Many thanks for all the kind things you say. It is a great help and encouragement to know I have given assistance and pleasure. As you admire my practicality will you please imitate it and tell me exactly how much money you can each afford to spend, as the route I should recommend would depend so much upon that. To say you want to do it cheaply does not convey a definite idea, because everyone's ideas as to cheapness are different. Give me the outside limit of your possible outlay which shall include the journey, and also tell me what your tastes are, whether they incline to inteiesting cities or to scenery most. I do not think it would be too cold on the Arlberg in June.

A Week in Paris (Christmas Rose).—It would have been better if you had told me what each of you could afford to spend, as there are hotels in Paris suited to almost all pockets. It is not a good plan in your circumstances to have inclusive terms, because, as you will spend your entire days sightseeing, you would almost certainly be far from your hotel at luncheon time on most days. Arrange to have your breakfast in the hotel and your late dinner, but take your luncheon at one of the many delightful restaurants that abound all over Paris. In that way you will waste neither time, money, nor temper. Very reasonable hotels and well placed are the following: Hôtel Brittanique, 20 Avenue Victoria; terms from about 8 frcs. per day. Hôtel Burgundy, in the Rue Duphot; Hôtel de l'Arcade, in the Rue de l'Arcade; Hôtel de Londres et de Milan, 8 Rue St. Hyacinthe. I have no space this week to give you a list of the places to see, but will do so next week. In the meantime if there is anything else you want to know write to me again.

In the Killarney District (I. H. W).—If economy be an object go in Dublin to the Edinburgh Temperance Hotel, 56 Upper Sackville Street, or Jury's Hotel, 7 College Green. From Dublin go to Athlone. By careful arrangement I do not think you need sleep at Athlone and it is not a very interesting place in itself. If a stay is necessary go to the Prince of Wales Hotel. Then take steamer down to Killaloe—a lovely trip not expensive, about 5s.—go to Grace's Hotel. From Killaloe go to Limerick and then on to Tralee. I do not think there is a better way of approaching Killarney than this for you. At Tralee, if necessary, stay at the Hôtel Central in Denny Street. At Killarney itself I should go if possible to O'Sullivan's Hotel in Muckross or in the town itself to M'Cowen's Temperance Hotel. In returning to Dublin I think you might work up by Mallow, Maryborough, and thence to Dublin.

Brittany and Normandy (Summer holiday).—Write me again how much you can afford to spend each, and I will draw you out a tour. The Normandy coast is not pretty. I should, in your place, spend all the time in Brittany. You could not see both properly in a month.

Rules in Regard to Correspondence for this Section.—All questioners must use a pseudonym for publication, but the communication must also bear the writer's own name and address as well, which will be regarded as confidential. All such communications to be addressed "Travel Correspondent, 28 Southampton Street, Strand." No charge will be made for inserting and answering questions in the inquiry column, and all will be answered in rotation as space permits. If an answer by letter is required, a stamped and addressed envelope must be enclosed, together with 2s. 6d., which fee will be devoted to the objects of "The Hospital" Convalescent Fund. Ten days must be allowed before an answer can be published.

### District Nursing: Typhoid.

BY A SUPERINTENDENT OF DISTRICT NURSES.

THE household was one of the jealously self-respecting and independent poor to which it is exceedingly difficult to find an entrance, and the case was in its fifth or sixth day before the doctor, realising that the mother was incapable of the work and was endangering the rest of the family, broke down the artificial barriers by giving us a character for kindness, zeal, discretion, and, above all, uniform success, that no nurses could possibly deserve.

A pleasant-looking woman received us, and said that the patient was her youngest child, a girl of seven.

We took temperature, pulse and respiration, and disinfected the thermometer. A temperature chart was begun, and a report of the patient's condition written for the doctor.

We next prepared to wash the patient. Finding that there was a discharge from her eyes we washed them, from the outer corner inwards with pieces of absorbent wool dipped in warm boracic lotion. As the mouth was covered with sordes we wrapped wool round the dressing forceps, dipped them in weak Condy and cleaned the teeth, tongue, and roof of mouth. We then let the child rinse her mouth with weak Condy followed by warm water. We asked the mother to buy a 2d. bottle of glycerine and borax and a soft 1d. gum brush before our evening visit.

We laid the child between two old blankets and the rest of the bedclothes were taken into the garden and hung on a line in the sun. Having been provided with a basin of warm water, a saucer with carbolic soap and flannel, two towels (one to be kept for the back), and a brush and comb, we took from our bag methylated spirit, dusting powder, nail scissors, and a piece of common wool on which to wipe them. Sponging had been ordered, but as the poor woman had been "afraid to put a drop of water near her flesh," the first sponging partook of the nature of an ordinary wash.

We first washed the face, neck and ears, and then sponged each limb for three minutes, sponging downwards with long straight strokes. The hands and feet we dipped in the basin, as it is refreshing to the patient, and helps to soften the nails before they are cut and cleaned. The abdomen we did not touch, but the back was sponged, and the back and both hips were carefully rubbed with methylated spirit and powdered. We explained this process and its object to the mother, and asked her to attend to the back herself at 2 P.M. and once in the night.

The doctor had ordered a pneumonia jacket, a child was sent to buy the materials, we cut it out, and while we sponged the patient the mother made it. We then put it on, and a clean night dress, and made the bed. As the counterpane was heavy we asked the mother to let us replace it by a sheet, and also to allow us to remove the valances, a heavy window curtain, the carpet, and all unnecessary furniture.

We drew the bed out from the wall, and placed under it a pie dish of boiling water with a teaspoonful of crude carbolic. (This may have no effect upon germs, but it reduces the smell, and the consequent nausea of mother and nurse).

A bed-pan had been lent, and we showed the mother how to make a cover for it out of an old piece of flannel, $1\frac{1}{2}$ yard long and 8 inches wide, with a string run through at both edges lengthways. We told her to put carbolic acid into all the discharges, never to allow them to remain under the bed, but to carry them to the w.c. at once, and to let them stand there, if possible, for half an hour before emptying them down the drain, and to lime-wash the w.c. every day. For a bed-pan cover we put a cloth into carbolic 1–40, begged the mother always to carry the bed-pan covered, and impressed on her the necessity of washing her hands with carbolic soap after attending to the patient. The necessary supply of carbolic was mixed and conspicuously labelled, and the woman emphatically warned to do nothing for the child at night without first turning up the lamp.

We then gave our attention to the family cups and saucers, of which we found five or six in the room. We explained how dangerous this was for the rest of the children, and the mother promised to put them all in a big saucepan filled with cold water and carbolic and let them boil. We cleaned the feeder that she had borrowed from a neighbour, and told her that it must be used exclusively by the patient, and that a special bowl and tea-cloth must be kept in the room and used when washing it.

There was no mackintosh, and we pondered how one was to be obtained, as the mention of the price called up lines of care on the poor woman's face. "How many brothers and sisters have you in the town?" I asked. "Seven," was the unexpected reply. "A square yard of mackintosh costs 3s. 6d. Suppose you ask each one of your brothers and sisters to give sixpence towards buying this most necessary article for their little niece?" In the course of two or three days the money was collected and the mackintosh bought, and every one of the seven aunts and uncles, and many of their friends and acquaintances, had learnt the price of mackintosh and its value as a nursing accessory.

We showed the mother how a Hinckes-Bird ventilator could be made, and she said that her husband had a holiday that afternoon and would be able to make it. In the meantime we arranged a substitute by opening the window at the bottom and filling up the space with a folded mat. As the day was warm, we left it open a little at the top.

In order to carbolise the room we asked for a long sweeping broom and a pail of water, to which two tablespoonfuls of crude carbolic had been added, and we soon did the floor, and dusted the room with a damp carbolised duster.

We told the mother that the child must have at least one quart of milk in 24 hours; that the milk must be boiled by standing it in a covered jug in a saucepan of boiling water; that the milk must be kept in the larder covered with a layer of wet muslin, and that only the amount immediately needed must be brought into the bedroom.

The nursing of every fever case should be started in the same way. The first visit is the most important one; after that everything goes smoothly and quickly. The next day the room is probably carbolised before the nurse arrives. When the poor have once been taught practically they seldom forget and slip back to a lower level. Recently we went in great haste to get a room ready for an operation, and to our surprise found it scrubbed out and everything clean. "How did you know what ought to be done?" we asked. "Oh!" was the reply, "I did same as what you done when my little boy had typhoid a year ago." The doctor had omitted to give us sufficient notice before the operation, and if the room had not already been clean it would have been physically impossible for us to get it ready in time.

Before leaving the mother we warned her on no account to allow the child to sit up in bed or to give her anything but milk, and impressed upon her the necessity of carrying out the doctor's instructions with absolute faithfulness and to the minutest detail.

The child made a good recovery with a shorter convalescence than is usual in typhoid, and no other cases occurred in the house.

### Notes and Queries.

**REGULATIONS.**

**The Editor is always willing to answer in this column, without any fee, all reasonable questions, as soon as possible.**

**But the following rules must be carefully observed.**

1. **Every communication must be accompanied by the name and address of the writer.**
2. **The question must always bear upon nursing, directly or indirectly.**

**If an answer is required by letter a fee of half-a-crown must be enclosed with the note containing the inquiry.**

*Homes.*

(10) I should be glad to know of any home where a child of two years, a girl, having no use in her legs, could be received. Parents are very poor, but would give a trifle towards maintenance if there is no free home available?—*Nurse J. F.*

Write to the Home for Incurable Children, Maida Vale, London, W., or see "Burdett's Hospitals and Charities," obtainable from the Scientific Press, Limited, 28 and 29 Southampton Street, Strand, W.C.

(11) Can you tell me of a home for aged females anywhere in or near Sheffield?—*E. L. B.*

St. Michael's Home for Aged Women, Liverpool, or see reply above.

*Nursing in Prisons.*

(12) Will you kindly tell me whether a nurse with ordinary general training is ever employed in a prison hospital? To whom can I apply for information?—*Dora.*

Write to the Prison Commissioner, Home Office, London, S.W.

*The Midwives Act.*

(13) Can you send me a copy of the Midwives Act? or tell me how or where to get it?—*C. S.*

You can obtain it from the Scientific Press, 28 & 29 Southampton Street, Strand, W.C. Price 2d. post free.

*Central Midwives Board.*

(14) I am the holder of the certificate of the London Obstetrical Society taken in 1900. Will you kindly tell me if it entitles me to the certificate of the Central Midwives Board?—*S. W.*

Yes, on certain conditions. Write to the Secretary, Central Midwives Board, 6 Suffolk Street, Pall Mall, S.W.

*Gynæcological Examination.*

(15) Will you kindly tell me where to write for particulars of the Gynæcological Examination?—*A. E. S.*

The Secretary, British Gynæcological Society, 437 Oxford Street, W.

*Nursing Abroad.*

(16) Can you give us any information on nursing abroad. Three of us are very anxious to go together?—*G. T.*

It all depends upon where you wish to go. For list of hospitals abroad see "Burdett's Hospitals and Charities."

*Institutions for Infants.*

(17) Can you tell me of an institution which would receive an infant four months old?—*Samaritan.*

Without further particulars it is impossible to answer this question.

*Dispensing.*

(18) Can you tell me how to get lessons in dispensing? Is it possible to join a correspondence class? Where can I apply for particulars?—*A. B. C.*

Write to Miss Buchanan, M.P.S., Gordon Hall, Gordon Square, W.C.

*Eye Bath.*

(19) Will you kindly tell me the right way to use an eye bath?—*E. G.*

This is a simple matter which the instrument maker from whom you purchase the bath would explain.

**Handbooks for Nurses.**

| | Post Free. |
|---|---|
| "A Handbook for Nurses." (Dr. J. K. Watson.) ... | 5s. 4d. |
| "The Nurses' Dictionary of Medical Terms." ... ... | 2s. 0d. |
| "Massage" (Swedish.) ... ... ... ... ... ... | 2s. 6d. |
| "Surgical Ward Work and Nursing." ... ... ... | 3s. 10d. |
| "A Complete Handbook of Midwifery." (Watson.) ... | 6s. 4d. |

Of all booksellers or of The Scientific Press, Limited, 28 & 29 Southampton Street, Strand, London, W.C.

### For Reading to the Sick.

THROUGH DARKNESS TO DAWN.

For as He bore the burden of the bitter Cross,
For as He died thereon, and dying did redeem—
So doth He bear thy burden, and thy name engross
Within the pages of the Book of Life—until it seem
Thou art a son indeed: no son shall suffer loss.

For what seemed loss to thee may end in greater gain—
Of conquest over self, and Truth made manifest;
Of that supremest Love, whose secret maketh plain
The meaning of thy life—and putteth to the test
Thy spirit in its reach and struggle to attain.

So in the faith that feareth not wait thou the dawn;
Knowing that in thy weakness shalt thou yet be strong—
Strong in His might who hath thy sin and weakness borne:
Soon shall the darkening shadows cease thy path to throng—
And daybreak open to the glad Eternal Morn!

When our friends betray us, and we ourselves are only too ready to deny Him; when, against us, evil gathers with its swords and its staves, and our soul is exceeding sorrowful, even unto death. Then He comes; He enters in; He abides; He sups with us; that we, His friends, may have peace. Peace! not from trouble, and anguish, and death; not the peace of easy safety; not the peace that the world longs after; but peace in Him who, amid all trouble, has pledged to us the victory; peace in that we possess within us Him who is stronger than all that can be against us. "Let not your hearts be troubled, neither let them be afraid. In the world ye shall have tribulation. But be of good cheer; I have overcome the world." *H. S. Holland.*

Take meekly the humiliations which God in His wise providence deals out to you; they are a most wholesome diet. They come from His hand who knows all that you need, who orders all in love, who bore the Cross for your redemption, and will, if you will let Him, heal your deep infirmities.

Place yourself often beneath the Cross of Calvary! see that sight of love and sorrow; hear those words of wonder; look at the Eternal Son humbling Himself there for you, and ask yourself, as you gaze fixedly on Him, whether he, whose only hope is in that Cross of absolute self-sacrifice and self-abasement, can dare to cherish in himself one self-exalting thought, or allow in himself one self-complacent action. *Bishop Wilberforce.*

May God give us grace not to value ourselves or any little sacrifices which He in His mercy may give us an opportunity of making for Him; but still to look forward to the unknown hour when we shall have to drink of His cup, to die as He hath died before us! With that hour full on our mind, may we cheerfully pass by whatever stands in our way, and, being called by Christ, give ourselves up forthwith to fulfil His holy commandments. *J. Keble.*

. . . . In that hour
From out my sullen heart a power
Broke, like the rainbow from the shower,
To feel, although no tongue can prove,
That every cloud, that spreads above,
And veileth love, itself is love. *Tennyson.*

